# A retrospective analysis of emergency surgery for cases of acute abdomen during cancer chemotherapy. Case series

**DOI:** 10.1016/j.amsu.2020.07.038

**Published:** 2020-07-23

**Authors:** Yoshiaki Maeda, Toshiki Shinohara, Nozomi Minagawa, Tadayuki Kobayashi, Ryota Koyama, Shingo Shimada, Yusuke Tsunetoshi, Keisuke Murayama, Haruka Hasegawa

**Affiliations:** Department of Gastrointestinal Surgery, Hokkaido Cancer Center, Japan

**Keywords:** Acute abdomen, Chemotherapy, Cancer chemotherapy, Emergency surgery, Anti-VEGF

## Abstract

**Background:**

Treatment for acute abdomen during chemotherapy is frequently difficult because of the complicated status of the patients, and there have been only a few case series summarizing the outcomes of emergent surgery during chemotherapy. The aim of this study was to clarify the clinical outcomes of emergency surgery for acute abdomen during chemotherapy and identify predictive factors associated with mortality.

**Methods:**

We retrospectively analyzed the records of patients who underwent emergency surgery for acute abdomen within 30-days after anti-cancer drugs administration between 2009 and 2020.

**Results:**

Thirty patients were identified. The primary malignancies were hematological (n = 7), colorectal (n = 4), lung (n = 4), stomach (n = 2), breast (n = 2), prostate (n = 2) and others (n = 5). Fifteen patients were treated with the regimen, including molecular-targeted anti-cancer drugs (Bevacizumab: 8 cases, Rituximab: 4, Ramucirumab: 2, and Gefitinib: 1). Indications for emergency surgery were perforation of the gastrointestinal tract (n = 24), appendicitis (n = 3), bowel obstruction (n = 2), and gallbladder perforation (n = 1). Severe morbidity (Clavien-Dindo IIIa or more) occurred in 8 cases (27%), and there were 6 in-hospital deaths (20%). Significant factors related to in-hospital death were age >70 years old (P = 0.029), poor performance status (ECOG score 1 or 2) (P = 0.0088), and serum albumin level <2.6 g/dl (P = 0.026). The incidence of acute abdomen (odds ratio 5.31, P = 0.00017) was significantly higher in the patients receiving anti-VEGF drugs than in those without anti-VEGF drugs.

**Conclusion:**

This study identified three predictive factors associated with in-hospital death after emergency surgery during chemotherapy: an older age, poor performance status, and low serum albumin level.

## Highlights

• Thirty patients underwent emergency surgery for acute abdomen after anti-cancer drugs administration.
• Mortality rate was 20%. Significant factors related to mortality were high age, poor PS, and low albumin level.
• The incidence of acute abdomen was significantly higher in the patients receiving anti-VEGF drugs.

## 1 Introduction

Recently, advances in chemotherapy, including molecular-targeted anti-cancer drugs, have greatly improved the prognosis and quality of life of patients with unresectable or recurrent cancer. With the development of more strong regimens including the combination of multiple anti-cancer drugs, the rates of severe adverse effects have been increasing, so treatment for complications related to chemotherapy is becoming important.

Acute abdomen, such as perforation of the gastrointestinal (GI) tract, is one of the most severe adverse events during chemotherapy. Treatment for acute abdomen during chemotherapy is frequently difficult because of the complicated status of the patients, such as the presence of severe neutropenia.

The present study clarified the clinical outcomes of emergency surgery for acute abdomen during cancer chemotherapy and identified predictive factors associated with surgical mortality. The incidence of acute abdomen during cancer chemotherapy was also estimated.

## 2 Patients and methods

We retrospectively analyzed the records of emergency surgery at our hospital between January 2009 and January 2020 using the database of gastrointestinal surgery division. Patients who underwent emergency surgery for acute abdomen within 30 days after anti-cancer drugs administration were included in the study. Background clinical factors, surgical procedures, and short-term outcomes were analyzed. Potential predicting factors associated with in-hospital death were evaluated. The number of chemotherapies administered during this period was counted using the database of the hospital's chemotherapy ordering system.

This study was approved by the ethics committee of the institute, and informed consent was obtained from the all presented patients. This work has been reported in line with the PROCESS criteria [1]. To investigate prognostic factors, a chi-square test was used to analyze the nominal variables. Statistical analyses were performed using the EZR statistical software program [2]. A value of P < 0.05 was considered to be statistically significant.

## 3 Results

### 3.1 Outcomes of emergent surgery during chemotherapy

**Fig. 1:**
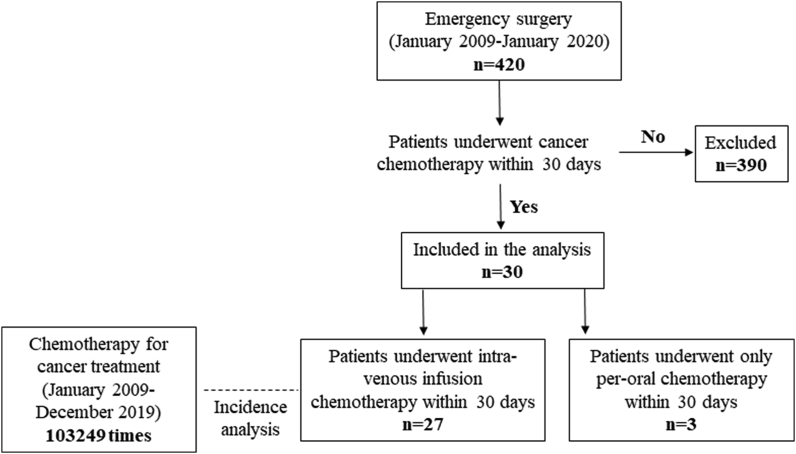
Overview of the patients included in the study.

Emergency surgery was performed for 420 cases in the gastrointestinal surgery division between January 2009 and January 2020. Thirty patients who had been receiving cancer chemotherapy within 30 days were included in this study. Twenty-seven patients had been receiving cancer chemotherapy associated with intra-venous anti-cancer drugs, and three had been treated by per-oral anti-cancer drugs. The number of chemotherapies performed in the relevant period was 103249, and these data were used to calculate the incidence of acute abdomen per treatment (Fig. 1).

The clinical characteristics of the patients are shown in Table 1. The group consisted of 15 women and 15 men, and the mean age was 64 years old (34–84). Primary malignancies were hematological (n = 7), colorectal (n = 4), lung (n = 4), stomach (n = 2), breast (n = 2), prostate (n = 2) and others (bladder, testis, uterus, pancreas, and soft tissue). Thirteen patients received multi-drug regimens consisting of ≥3 anti-cancer drugs, and 15 were treated with regimens including molecular-targeted anti-cancer drugs (Bevacizumab: 8 cases, Rituximab: 4, Ramucirumab: 2, and Gefitinib: 1).

**Table 1:** Demographics and clinical characteristics of the patients.

Age^a^	64 (34–84)	Chemotherapy regimen^c^	
Gender (F/M)	15/15	CBDCA + PEM + BEV	2
Primary cancer		FOLFOX + BEV	2
Hematological	7	TC + BEV	2
Colorectal	4	FOLFIRI + RAM	2
Lung	4	GEM + CDDP	2
Ovary	4	CHOP	2
Stomach	2	TC	2
Breast	2	DTX	2
Prostate	2	CDGP + BEV	1
Others^b^	5	PEM + BEV	1
Chemotherapy		RIT + ESHAC	1
Intravenous/per oral	27/3	RIT + TEMO	1
Anti-VEGF drugs (used/not used)	10/17	ADM + VCR	1
ECOG performance score (0/1/2/3)	20/8/2/0	IFO + ADM	1
ASA classification (1E/2E/3E)	15/13/2	GEM + L-OHP	1
BMI^a^	19.8 (16.8–37.2)	FEC	1
Time since cancer diagnosis (<3 months/3–12 months/1year <)	12/6/12	FOLFOX	1
		DCS	1
		PTX	1
Time since latest chemotherapy (<3 days/4–10 days/11 days <)	8/13/9	Fulvestrant + Abemaciclib	1
		Gefitinib	1
		Lenalidomide + Dexamethasone	1

a Mean + range.

b Bladder, testis, uterus, pancreas, and soft tissue.

c ADM Doxorubicin, BEV Bevacizumab, CBDCA Carboplatin, CDDP Cisplatin, CDGP Nedaplatin, CHOP CPA + ADM + VCR, CPA Cyclophosphamide, DCS Docetaxel + Cisplatin + S1, DTX Docetaxel, ESHAP Etoposide + Solumedrol + high-dose AraC + Cisplatin, FEC Fluouraci + Epirubicin Hydrochloride + Cyclophosphamide, GEM Gemcitabine Hydrochloride, IFO Ifosfamide, L-OHP Oxaliplatin, PEM Pemetrexed Sodium Hydrate, RAM Ramcizumab, RIT Rituximab, TC Paclitaxel + Carboplatin, TEMO Temozolomide, VCR Vincristine.

The profile of surgical procedures for acute abdomen and short-term results of the operation are shown in Table 2. Indications for emergency surgery were perforation of the GI tract (n = 24), appendicitis (n = 3), bowel obstruction (n = 2), and gallbladder perforation (n = 1). GI tract perforation occurred at the cancer site in 14 cases, and at other sites in 10 cases. The operative procedures performed were resection (n = 7), stoma (n = 5), omentum patch (n = 4), resection and stoma (n = 3), drainage only (n = 3), appendectomy (n = 3), patch and stoma (n = 2), bypass (n = 2), and cholecystectomy (n = 1). The median operative time was 85 min (range 55–165), and the median blood loss was 10 ml (range 0–2750). Severe morbidity (Clavien-Dindo ≥ IIIa) occurred in 8 cases (27%), and there were 6 in-hospital deaths (20%).

**Table 2:** Operative procedures performed for acute abdomen after chemotherapy.

Character of acute abdomen	
GI perforation	24
Appendicitis	3
Bowel obstruction	2
Gallbladder perforation	1
Site of GI perforation	
Cancer site/Others	14/10
Operative procedure	
Resection	7
Stoma	5
Omentum patch	4
Resection + stoma	3
Drainage only	3
Appendectomy	3
Omentum patch + stoma	2
Bypass	2
Cholecystectomy	1
Operative time (minutes)^a^	85 (55–165)
Blood loss (ml)^a^	10 (0–2750)
Hospital stay (days)^a^	25 (10–155)
Morbidity	
Clavien-Dindo 0/I/II/III/IV/V	9/2/9/6/2/2
Mortality	
30-day mortality	2
In hospital death	6

a Median + range.

The results of a univariate analysis for potential factors predicting in-hospital death among the cases of acute abdomen during chemotherapy are shown in Table 3. Significant factors related to in-hospital death were an age >70 years old (odds ratio 8.9, P = 0.029), poor performance status (PS; ECOG score 1 or 2) (odds ratio 16.7, P = 0.0088), and serum albumin level <2.6 g/dl (odds ratio 11.1, P = 0.026).

**Table 3:** Factors associated with in-hospital mortality after emergency surgery for acute abdomen after chemotherapy.

	Alive	In-hospital death	Odds ratio	95% CI	P value
	(n = 24)	(n = 6 =			
Age					
<69 years old	20	2	8.9	0.94–132	0.029
70 years old<	4	4			
Gender					
Female	11	4	–	–	N.S.
Male	13	2			
Primary cancer					
Hematological	6	1	–	–	N.S.
Colorectal	3	1			
Lung	3	1			
Ovary	2	2			
Others	10	1			
Chemotherapy					
Intravenous	22	5	–	–	N.S.
Per oral drug only	2	1			
Chemotherapy					
Single drug	3	1	–	–	N.S.
2 drugs	11	2			
3 or more drugs	10	3			
Anti-VEGF drugs					
Used	7	3	–	–	N.S.
Not used	17	3			
ECOG performance score					
ECOG 0	19	1	16.7	1.14–937	0.0088
ECOG 1–2	5	5			
ASA classification					
1E	14	1	–	–	N.S.
2E or 3E	10	5			
Time since cancer diagnosis					
<3 months	10	2	–	–	N.S.
3–12 months	5	1			
12 months <	9	3			
Time since latest chemotherapy					
<3 days	8	0	–	–	N.S.
4–10 days	4	4			
11 days <	7	2			
Character of acute abdomen					
GI perforation	18	6	–	–	N.S.
Others	6	0			
Morbidity					
Clavien-Dindo 0-II	17	3	–	–	N.S.
Clavien-Dindo III <	7	3			
Albumin					
<2.6 g/dl	7	5	11.1	0.99–604	0.026
2.6 g/dl<	17	1			
Total protein					
<5.0 g/dl	5	3	–	–	N.S.
5.0 g/dl<	19	3			
Hemoglobin					
<10 g/dl	7	2	–	–	N.S.
10 g/dl<	17	4			
Cholinesterase					
<150 U/l	11	5	–	–	N.S.
150 U/l<	13	1			
WBC					
<3000/mm3	8	3	–	–	N.S.
3000–10000/mm3	10	2			
1000/mm30<	6	1			
Neutrophil					
<1500/mm3	5	0	–	–	N.S.
1500/mm3<	16	3			

N.S. No statistical significance.

### 3.2 Incidence of acute abdomen and GI perforation during chemotherapy

In the study period, 103249 chemotherapies, including intra-venous infusion, were performed, so the incidence of acute abdomen needing surgery was 0.026% (27/103249) per therapy session. Anti-VEGF drugs were used in 10311 chemotherapies (Bevacizumab: 8248, Ramucirumab: 1285, Panitumumab: 784, and Aflibercept Beta: 30). The incidence of both acute abdomen (odds ratio 5.31, P = 0.00017) and GI tract perforation (odds ratio 6.62, P = 0.00011) was significantly higher in the patients receiving anti-VEGF drugs than in those without anti-VEGF drugs (Table 4).

**Table 4:** Impact of the administration of anti-VEGF drugs for incidence of acute abdomen.

		Emergent surgery for acute abdomen				
		(+)	(-)	Incidence	P value	Odds ratio
Anti-VEGF drugs	Used	10	10301	0.097%	0.00017	5.31
Not used		17	92921	0.018%		

		Emergency surgery for GI perforation				
		(+)	(-)	Incidence	P value	Odds ratio
Anti-VEGF drugs	Used	9	10302	0.087%	0.00011	6.62
Not used		12	92926	0.013%		

## 4 Discussion

An oncologic emergency is an acute condition of a cancer patient that develops directly or indirectly from cancer or cancer treatment. Acute abdomen is one of the most severe oncologic emergencies and includes GI perforation, GI obstruction, appendicitis, and others. Patients developing acute abdomen as a symptom of oncologic emergencies can typically only be rescued by surgical treatment; however, the surgical mortality rates after emergency surgery for oncological emergencies, such as perforated GI, have been reported to be very high, ranging from 11% to 42% [[3], [4], [5], [6]]. Some authors have reported predictive risk factors for mortality after surgery for oncologic emergency [[6], [7], [8], [9]]; however, the literature describing the outcome of emergency surgery for patients receiving cancer chemotherapy is extremely limited [10]. Since the time for decision-making is limited due to the emergency status of the patients, there is a need for objective parameters that assist in predicting the outcome of surgical intervention for acute abdomen during chemotherapy.

In the present study, we identified three predictive factors associated with in-hospital death after emergency surgery during chemotherapy: an age >70 years old, poor PS (ECOG >0), and serum albumin level <2.6 g/dl. It is natural that an older age was identified as a negative factor related to in-hospital death, since an older age has been reported to be a poor prognostic factor associated with oncologic emergency in many reports [7,11]. A poor PS has also been reported to be a strong poor prognostic factor for not only the surgical outcome for oncologic emergency [6,8] but also the outcome of chemotherapy itself [[12], [13], [14]]. Most clinical trials of chemotherapy include patients with a good PS only [15]; however, in the real world, cancer patients with a poor PS often undergo chemotherapy. The present study clearly showed that a poor PS was a risk factor for mortality after emergency surgery during chemotherapy. A low serum albumin level has also been reported to be a poor prognostic indicator for the surgical outcome in patients associated with oncological emergency [8,9]. The serum albumin level has been identified as a significant prognostic factor for patients with various types of cancer [[16], [17], [18]]. This reflects the important role of serum albumin as a biomarker of the visceral protein and immunocompetence status, which is fundamental for the biological nutritional assessment [19]. In the present case series, in-hospital mortality rate of the patients who had all 3 of these risk factors was 75%. Considering the poor prognostic factors related to in-hospital death identified in this study, special care should be taken when administering chemotherapy to cancer patients who are elderly or have a poor PS or poor nutrition status.

The present study also showed that the incidence of both acute abdomen and GI tract perforation was significantly higher in patients receiving chemotherapy with anti-VEGF drugs than in those not being treated with anti-VEGF drugs. Anti VEGF agents, such as bevacizumab, ramucirumab, panitumumab, and aflibercept beta, inhibit neovascularization in the tumor tissue and can delay tumor growth [20]. A stronger response has been shown by the combination of conventional chemotherapy and anti-VEGF agents in various types of cancer. Indeed, guidelines around the world recommend the combination of anti-VEGF agents and chemotherapy as an option for treatment of many cancers, including colorectal, lung, and ovarian cancer [21]. However, while a high efficacy of anti-VEGF agents has been reported, serious adverse effects have also been described, including arterial thrombosis, hemorrhaging, and GI perforation. Many clinical trials of anti-VEGF drugs have shown that patients receiving anti-VEGF drugs had higher rates of GI perforation than those without such treatment [[21], [22], [23], [24], [25]]. Several authors have further reported that the risk of emergency surgery due to anti-VEGF agent-related severe adverse effects in advanced cancer was estimated to be as high as 2.8% [23,[26], [27], [28], [29]]. Other authors reported that the fatality rate of patients with GI perforation treated with anti-VEGF drugs was as high as 20% [30].

There have been many case reports of acute abdomen including GI perforation in patients with various kinds of cancer associated with chemotherapy [[31], [32], [33], [34], [35], [36], [37]]; however, there have been only two reports summarizing surgery cases of acute abdomen during cancer chemotherapy [10,38]. To our knowledge, this is the first report to clarify the risk factors for mortality after emergency surgery for acute abdomen during cancer chemotherapy.

Several limitations associated with the present study warrant mention. This was a retrospective analysis performed at a single hospital with a limited number of patients, including heterogenous patients with various cancer types and receiving various chemotherapy regimens. Since this was not a prospective study and was based on the database of the surgery branch, we might have missed cases not referred to surgeons who received best supported care only. The incidence of acute abdomen among patients treated by per-oral anti-cancer drugs only was also not clarified in this study. A prospective study including a larger patient number will be necessary to establish a guideline for the treatment of patients with acute abdomen related to chemotherapy.

## 5 Conclusion

This study identified three predictive factors associated with in-hospital death after emergency surgery during chemotherapy: an age >70 years old, poor PS (ECOG >0), and serum albumin level <2.6 g/dl. Furthermore, the incidence of GI tract perforation during chemotherapy was approximately six times higher in the patients receiving anti-VEGF drugs than in those without anti-VEGF drugs. Clinicians should take these risk factors into consideration when performing cancer chemotherapy.

## Statement of ethics

The authors have no ethical conflicts to disclosure. This study was approved by ethical committee of the institute, and informed consent was obtained from the all presented patients.

## Funding

This study was not supported by any grant or funding.

## Ethical Approval

This study was approved by the ethical committee of the hospital (Approved No. 30-71)

## Consent

Anonymity of the patients has been strictly protected in the present study.

## Author contribution

Study conception and design: Maeda, Shinohara

Acquisition of data: Maeda, Shinohara, Koyama, Kobayashi, Murayama, Hasegawa

Operator of surgery: Maeda, Shinohara, Minagawa, Shimada

Drafting of manuscript: Maeda, Shimada, Minagawa, Tsunetoshi.

## Registration of Research Studies

Name of the registry: UMIN-CTR

Unique Identifying number or registration ID: UMIN 000040315

Hyperlink to your specific registration (must be publicly accessible and will be checked): https://upload.umin.ac.jp/cgi-open-bin/ctr/ctr_view.cgi?recptno=R000045997

## Guarantor

Yoshiaki Maeda

## Provenance and peer review

Not commissioned, externally peer reviewed.

## Declaration of competing interest

The authors have no financial interests or potential conflicts of interest.
